# Childhood Obesity Trends among 8–11-Year-Olds: Insights from a School Sample in Vienna, Austria (2017–2023)

**DOI:** 10.3390/children11040431

**Published:** 2024-04-03

**Authors:** Paula Moliterno, Victoria Donhauser, Kurt Widhalm

**Affiliations:** 1 Austrian Academic Institute for Clinical Nutrition, 1090 Vienna, Austria; paula.moliterno@eddykids.at (P.M.); donhauser@eddykids.at (V.D.); 2 Division of Clinical Nutrition and Prevention, Department of Pediatrics, Medical University of Vienna, 1090 Vienna, Austria

**Keywords:** obesity, childhood, trend, Austria, COVID-19

## Abstract

In Austria, childhood obesity is a public health concern. This study examined time trends in the percentage of obesity among a sample of schoolchildren from Vienna (2017–2023). The body mass index percentiles of 326 children [9.3 years old (95% CI 8.3–10.5, min–max 8.0–10.9] from the EDDY study were calculated for trend analyses. Trend analysis was performed using a logistic regression using overweight and obesity as dependent binary variables, and study year and age as independent continuous variables. The percentage change over time was calculated, including the first period of the COVID-19 pandemic. Obesity percentages increased from 23.5 (95% CI 15.1–31.9)% in 2017 to 25.0 (95% CI 12.2–37.8)% in 2023. From 2017 to 2023, while overweight/obesity percentages decreased by 25.9 (−59.5–15.6)%, obesity increased by 6.4 (−51.2–94.9)%. A non-significant trend (*p* ≥ 0.38) was observed. During the first period of the pandemic, the percentage changes in overweight/obesity and obesity were 68.4 (5.6–187.9)% and 29.2 (−37.3–166.8)%, respectively. The current percentage of obesity in this sample is high and peaked in 2020 during the pandemic. These findings emphasize the need for future investigations considering the representativeness of the school-aged population in Austria to gain a broader picture of overweight and obesity trends.

## 1. Introduction

It has been recently reported that between 2020 and 2035, the prevalence of childhood obesity is expected to increase globally by 100% [1]. In Austria, a high childhood obesity risk score has been assigned according to the World Obesity Federation [2] through a list of indicators that include the percentage of boys and girls with obesity and insufficient physical activity, among others. As in other European countries, childhood obesity rates are of public health interest [3]. Although the overall prevalence of childhood overweight and obesity remained stable from 2016 to 2019, following the first and second Childhood Obesity Surveillance Initiative (COSI) rounds by the World Health Organisation (WHO), 29.5% of boys and 24.4% of girls from 7 to 10 years old have overweight or obesity [4]. Moreover, the World Obesity Federation has recently predicted that by 2030, 169,212 children aged 10–19 in Austria will live with obesity [2].

Children with excessive body weight for their age present a higher probability of excessive weight during adulthood [5], leading to a higher risk of comorbidities that suppose greater health costs and a negative impact on longevity [6]. A previous study in Vienna has shown that boys with overweight or obesity at a young age present a higher risk of these conditions at ages 10 and 15 [7]. Moreover, a large cohort including adolescents from Austria has shown that more than 50% of adolescents with obesity had at least one risk factor for cardiovascular disease, with elevated blood pressure being the most frequent, present in 35.4% of them [8]. In terms of worldwide trends, since 1975, the prevalence of obesity has increased from 0.7% (0.4–1.2) to 5.6% (4.8–6.5) in 2016 in girls, and from 0.9% (0.5–1.3) to 7.8% (6.7–9.1) in 2016 in boys [9]. Some reports have even shown a plateau in rising trends since the year 2000 [9,10] with a stabilization in the prevalence of overweight and obesity [11,12], at least until the up-rise of the COVID-19 pandemic, when an acceleration in children’s BMI and obesity rates occurred, as regular lifestyle was disrupted [13,14]. During the latter period, overall, school-age children’s body weight and body mass index (BMI) increased (mean differences of 2.67 kg and 0.77 kg/m^2^, respectively) despite the heterogeneity of studies [13]. In Austria, a higher increase in body weight was reported in school-aged children during the lockdown period compared to the previous year [15]. Acknowledging all factors that mediate obesity during childhood while planning prevention interventions is essential to determine the effectiveness of the interventions [16].

Time trend analysis on the percentage of overweight and obesity among a sample of schoolchildren may be of interest to support health actions to tackle this problem; moreover, this type of information is still scarce in Austria. Therefore, we aimed to examine time trends in the percentage of overweight and obesity in a sample of schoolchildren from Vienna, Austria, during 2017–2023.

## 2. Materials and Methods

### 2.1. Sample

The present study included a total of 326 children (aged ≥ 8, <11 years) from 2017 to 2023, with measures of height and weight in a sample from an elementary school in Vienna, Austria, located in the 12th district. Regarding the district’s socioeconomic status, the average net income falls within the middle range among Vienna’s districts; however, it is lower than the national average. The percentage of unemployed individuals is high compared to the Vienna average. Although in terms of education, the neighborhood has a low proportion of academics compared to other Vienna districts, in the national context, the proportion of individuals with academic qualifications is higher [17]. 

Each year, a non-randomized sample of students from 3rd grade who were willing to participate was included. The sample size was 98 in 2017, 64 in 2018, 46 in 2020, 30 in 2021, 44 in 2022 and 44 in 2023. The present analysis focused on tracking and analyzing trends in overweight and obesity within the elementary school setting, so we restricted the analysis to a specific class to provide a more homogeneous group without necessitating a consistent, unchanged population sample over the study’s duration. Therefore, this study does not include linked samples of participants. The sample belonged to the EDDY (“Effect of sports and diet training to prevent obesity and secondary diseases and to influence young children’s lifestyle”) study, an intervention study aiming to improve nutrition and physical activity habits by imparting a six-month educational training in 4th-grade students [15]. The EDDY study was initiated by the Austrian Academic Institute for Nutrition in Vienna, Austria, in collaboration with the Institute for Sports and Movement Science at the University of Vienna. For the intervention group, the educational training was seamlessly integrated into the school curriculum as part of the regular program. On the other hand, the 3rd grade classes that acted as the control groups followed the school curriculum, where there was no dedicated intervention to promote healthy habits.

Approval for the study’s ethical considerations was obtained from the Ethical Committee of Sigmund Freud University, Vienna (PAFGRW9O@EFQV885378–15 September 2016). Before each study edition, the EDDY team introduced and explained the project to the parents and asked them to provide written informed consent to participate. Children themselves gave their consent by using the assent form. All data collected underwent an anonymization process, ensuring that individual identities were dissociated from the information gathered. Participation was voluntary, and no compensation was provided. The ability to drop out was provided at any point. The present analysis included, for each investigation year, children from the 3rd grade classes with complete data for the dependent variables. Anthropometric measurements were conducted within the school setting in September 2017, December 2018, June 2020, 2022, 2023, and February 2021. Measurements comprised the same seasonal period, except for the years 2020 and 2022, where outbreaks of the COVID-19 pandemic disturbed the normal development of the study, and in 2023, due to a change in the methodology of the study. The differences in the general characteristics between those participants included in this study and those excluded are shown in Supplementary Material, Table S1. 

Children included in this analysis were younger than those who were not included (*p* < 0.00010). However, the proportion of female participants and BMI values was the same (*p* > 0.060). The sexual categorization of participants was determined by the research team through the external observation of bodily secondary sexual characteristics such as physical appearance, and discerning whether each child presented characteristics indicative of male or female sex. Migration background data of the children were unavailable for this study.

### 2.2. Anthropometric Measurements

Trained technicians measured body weight in kilograms using a Tanita body composition electronic scale (MC-780MA, TANITA Corporation, Tokyo, Japan), and height in centimeters using a portable stadiometer (SECA 213, Hamburg, Germany), following standardized methods. All measurements were performed in the school. Children were advised to wear light indoor clothes and be barefoot, and each participant’s measurements were performed in private. During the COVID-19 pandemic period, direct measurements were also performed. BMI was then calculated (weight/height^2^–kg/m^2^), and BMI-for-age percentiles were used to classify nutritional status using the German national reference criteria by Kromeyer-Hauschild et al. [18]. The latter is part of the standard guidelines of the Working Group of Obesity in Childhood and Adolescence (Arbeitsgemeinschaft Adipositas im Kindes- und Jugendalter; AGA), of the German Society of Obesity (Deutsche Adipositasgesellschaft). Low weight was classified as <3rd percentile, normal weight ≥ 3rd percentile and overweight ≥ 90th percentile. For statistical power purposes, the obesity category was considered as the sum of the original categories of obesity (≥97th percentile) and extreme obesity (≥99.5th percentile) [18]. Throughout the article, “overweight including obesity” was specified as overweight/obesity.

### 2.3. Statistical Analysis

Normality tests were carried out using the Shapiro–Wilk test. Descriptive variables are presented as absolute numbers and percentages or the median and 95% confidence interval (95% CI). Cross-sectional percentages of children with overweight/obesity and obesity were calculated for each year studied, for the whole sample and by sex. The differences in median BMI during the studied years were calculated using the Kruskal–Wallis test. Trend analysis was performed using logistic regression using overweight/obesity and obesity as dependent binary variables, and the investigation year and age as independent continuous variables. Finally, to study how the percentage of childhood overweight/obesity and obesity changed over time, we divided the difference between the percentage in 2023 and the percentage in 2017 by the percentage in 2017 and multiplied by 100. Moreover, to explore changes during the COVID-19 pandemic period, we additionally calculated the percentage change over time from 2018 to 2020. This study chose the peri-pandemic period starting in 2018 to incorporate available data closer to the onset of the COVID-19 pandemic.

Data analysis was conducted using SAS OnDemand for Academics (Cary, NC, USA), and a *p*-value < 0.050 was assigned as significant.

## 3. Results

A total of 326 children were included in the analysis (44.5% were female). Of them, 33.4% were 8 years old, 53.1% were 9, and 13.5% were 10. In general, 16.0% of the children had overweight and 22.1% of the children had obesity. The general characteristics of the participants by year of investigation are shown in Table 1.

The median (95% CI) BMI in 2017 was 19.3 (95% CI 14.7–28.5) kg/m^2^, and in 2023, it was 17.5 (95% CI 14.3–27.0) kg/m^2^, while in 2020, during the peri-pandemic period, the registered median BMI was 20.3 (95% CI 15.2–28.2) kg/m^2^. During the studied years, no statistical differences were observed in the median values of BMI for the overall sample (*p* = 0.069).

Throughout the years, the percentage of children aged 8 years with obesity was 22.0 (95% CI 14.2–29.8)%, while for those aged 9 years, it was 21.4 (95% CI 15.3–27.5)% and for those aged 10 years, it was 25.0 (95% CI 12.2–37.8)%. Distribution by sex can be observed in Table S2 (Supplementary Material, Table S2). 

From 2017 to 2023, obesity percentages changed from 23.5 (95% CI 15.1–31.9)% to 25.0 (95% CI 12.2–37.8)%, while the sum of overweight and obesity decreased from 42.9 (95% CI 33.1–52.7) in 2017 to 31.8 (95% CI 18.0–45.6)% in 2023 (Table 2).

**Figure 1:**
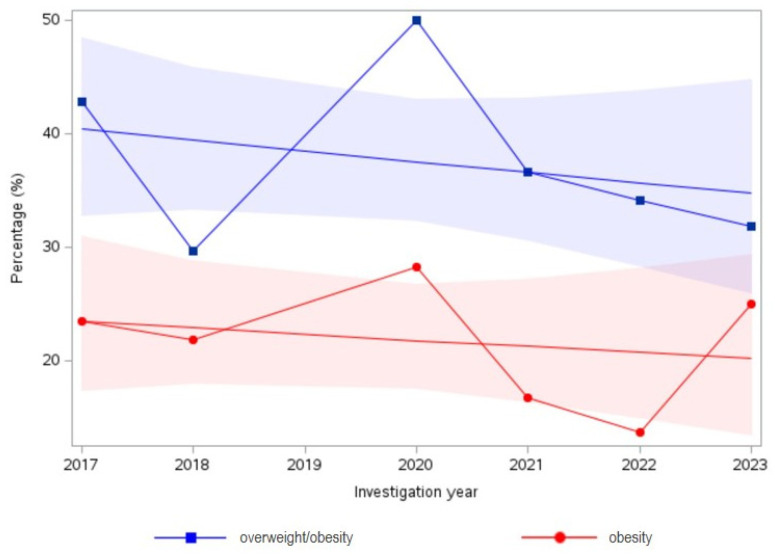
Percentage (%) of overweight and obesity according to study year and linear trends with the 95 % CI (shaded). *p* = 0.38 for overweight/obesity and *p* = 0.61 for obesity.

Table 2 shows the percentage of overweight/obesity and obesity over the six years studied. Assessment showed that the trend of percentage of overweight/obesity (*p* = 0.38) and obesity (*p* = 0.61) was not significant throughout the years (Table 2). Visual inspection of the data can be seen in Figure 1. 

When analyzed by sex, in female children, the percentage of overweight/obesity changed from 27.3 (95% CI 14.1–40.5)% in 2017 to 33.3 (95% CI 12.9–53.1)% in 2023 (Supplementary Material, Table S3). In boys, the percentage of overweight/obesity was 55.6 (95% CI 42.3–68.9)% in 2017; by 2023, it was 30.4 (95% CI 11.6–49.2)%. In no case was the trend of the percentage of overweight/obesity and obesity significant throughout the years (*p* ≥ 0.082) (Supplementary Material, Table S3).

Throughout the studied period (2017–2023), the percentage of change in overweight/obesity was −25.9 (−59.5–15.6)%, while the change in obesity rate was 6.4 (−51.2–94.9)% (Table 3). 

Analysis including the peri-pandemic period showed that the percentage of overweight/obesity and obesity increased by 68.4 (−51.2–94.9)% and 29.2 (−37.3–166.8)%, respectively (Table 3). In both female and male children, the percentage of obesity during the studied peri-pandemic period increased by around 30% (Table 3).

## 4. Discussion

The main findings of our study were the non-significant trends in the percentage of obesity in a sample of Viennese schoolchildren from 2017 to 2023, although it is worth noting that the percentage of overweight and obesity remains unacceptably high, with almost one-third of the children in this sample living with excessive body weight for their age. In this current analysis, although not statistically significant, the highest percentage of overweight and obesity was achieved in 2020, during the peri-pandemic period, mainly related to excessive rates in boys. 

From 2017 to 2023, in this sample, obesity percentages remained roughly between 22 and 25% (except for the peri-pandemic period), with a non-significant trend. Previous reports have shown a plateau or even decline in the rate of childhood obesity in some developed countries since the year 2000 [10]. Moreover, an international analysis including population-based studies with more than 500,000 children from nine countries reported stabilization in the rates of childhood obesity [11], occurring at different prevalence levels across the periods reported, ranging from 13.5% in France to 37.4% in the USA [11]. 

Several reasons have been proposed for the observed trends in childhood obesity, such as individual behavior changes related to environmental and social changes that affect the whole population, and therefore claimed for public health initiatives [10]. One possible reason explaining the non-significant trend in our study may be the several national initiatives implemented in Austria to promote healthy habits among children [19]. Since 2012, the promotion of physical activity in everyday life has been settled as a national health goal. This involves not only adjusting living environments in the cities (such as cycle paths, playgrounds and school routes) in a way that enables and encourages exercise, but also promoting exercise skills and enjoyment in exercise and sports in kindergartens and schools. In 2013, the National Action Plan on Physical Activity (NAP.b) was published, contributing to the latter aims. The initiative “Healthy Exercise for Children 2.0” [20], funded by the Federal Ministry of Public Service and Sport and coordinated by Fit Sport Austria, allows kindergartens and primary schools to take advantage of free exercise programs and sports clubs run by qualified exercise instructors. Initiatives like the latter are important, as the last COSI report observed that children in Austria who are members of a sports club or who play actively for several hours a day are significantly less likely to be overweight [4].

In 2011, the Federal Ministry of Health and Women’s Affairs published a National Action Plan for Nutrition (NAP.e) with a complete set of strategies and activities in the field of nutrition, and a close link with the health targets for Austria published a year after. Since then, evidence-based recommendations have been adopted to address different risk factors of obesity, such as nutrition during pregnancy and meals in kindergartens and schools. Also, the REVAN program has been launched as a health promotion program to improve nutrition, which also addresses a set of measures for children between the ages of 4 and 10 years [21]. Among these measures, the initiative “Children Eat Healthy” by the Healthy Austria Fund aims to promote and implement measures and projects nationwide to improve children’s nutrition within primary schools and communities. It began a pilot phase from 2020 to 2023 in four federal states. The evaluation showed positive improvements in primary school children’s eating habits and nutritional knowledge, as well as parents’ nutritional knowledge and the ability to talk to children about healthy eating [22]. In 2024, the program is entering its second phase, where a healthy and environmentally friendly diet remains a crucial focus. Another initiative implemented in the school setting since 2014 is the Vienna School Fruit Program, as in all public compulsory schools in Vienna. The Vienna School Fruit Program aims to teach children the importance of healthy and regional foods, and provide more knowledge about their origins. Lastly, it aims to foster enjoyment in fruits and vegetables at an age when eating habits are being formed. This project, funded by the European Union, distributes up to 20 tons of free fruits or vegetables to 100,000 pupils at compulsory schools every week and organizes tasting workshops. Although successful, an evaluation of the health promotion project to ensure that the intended goals are achieved and assess the effectiveness of the measures implemented is currently lacking [23]. Moreover, the best practice models have been identified as part of the strategies to tackle non-communicable diseases, involving food-based dietary guidelines with a comic graphical illustration for children and the integration of nutritional education into curricula [21]. 

On the other hand, although initiatives have been comprehensive, only 5% of the schools have reported having projects related to healthy lifestyle topics [4], showing that collective efforts still need to be completed, along with surveillance measurements, as it has been stated that there is currently insufficient data to assess whether some progress has been made to prevent an increase in obesity rates [24]. Although in Austria, every child entering the school system should be measured and their weight and height collected by school physicians, comprehensive numbers from schools are only partially available. Moreover, as shown by the first and second COSI rounds, children’s participation in studies has proven difficult, with the levels of participation (by parents’ agreement) of 49.3% and 44.2%, respectively [3,4]. 

Some authors have attributed parents’ decline in participation of their children in measurements involving body weight in cases where their kids are living with overweight or obesity because of stigmatization [11]. This self-selection bias could also possibly explain the stabilization trends in obesity reported previously; however, self-selection bias is unlikely to occur in the case of our study, as it was school-settled. In order to help improve monitoring data, a software (wachstum.at Accessed on: 20 February 2024) for assessing body measurement data in children is intended to be implemented nationwide with support from the Austrian Society for Paediatrics and Adolescent Medicine [25]. Considering the difficulty in participation in studies, using the software as a decision-making aid [25] could narrow some of the existing gaps in monitoring the evolution of children’s BMI as a surrogate of the nutritional status, although not at a population-based level. On the other hand, to define and study the change in the prevalence of overweight and obesity in children in Austria, referencing tools to assess BMI, such as Kromeyer-Hauschild and collaborators’ charts [18], the national reference values proposed by Mayer and collaborators [26], or the WHO BMI charts, should be consistent to allow for prevalence estimates to be comparable. 

Recent estimates from the World Obesity Federation suggest that the chance for Austria to meet the 2025 WHO target of no increase in 2010–2012 obesity levels is 11% [2]. The latter may suggest that there may be a need for more targeted interventions. Obesity during infancy is a complex phenomenon that involves lifestyle, socioeconomic status, social relationships and migration background, among others [27,28]. Considering that already a third of the boys and a quarter of the girls in Austria are affected by excessive body weight [4], this implies the need to implement treatment in the short and long terms. Once settled, obesity treatment during childhood is difficult, and in order to be effective in reducing weight loss and improving risk factors such as cardiometabolic parameters, it should comprise a multi-component approach [29]. An example of a multi-component healthy weight management program that is elementary school-based is the SNAPSHOT trial [30]. This program, targeting 8- to 12-year-old children, aims to reduce excess weight gain by increasing healthy dietary practices and physical activity, and decreasing sedentary behaviors. The nine-month intervention, school-nurse-led, includes parents and is delivered during out-of-school time, including four home visits with the parent/child dyad and two monthly contacts. Moreover, the kids and the parents participate in supportive groups with peers and receive a monthly newsletter with healthy information for the family. Such a model of secondary obesity prevention would be attractive to evaluate in the Austrian context considering the facilitators available in the school system, such as the school doctor and the familiar, safe school space.

The higher percentages of excessive body weight found in our study compared to national estimates could be related to the cultural background. Although migration background in this study was not available, the neighborhood where the school from this study was carried out presents a 49.8% migration background, higher than the overall average 44.4% estimate for Vienna [31]. 

Moreover, it has been reported that 46% of schoolchildren from Vienna have a migration background [32]. Feeding practices and societal ideals around body size may be strongly driven by cultural variances [16]. It is important to note that more than a quarter of the Austrian population has a migration background [33], so differences in cultural norms, socioeconomic circumstances and parental education [6] related to having a migration background may be the possible reasons influencing these differences. A previous report in a representative Viennese sample of 24,989 children and adolescents observed that having a migration background (assessed by having another mother tongue than German as a surrogate) was associated with higher obesity prevalence rates [32]. The latest COSI report showed that the highest prevalence of obesity (5.5%) was observed in boys living in the east region of the country, where Vienna is located [4]. 

Nonetheless, our results exceed the obesity rates found in the country’s western region [4], which may imply that Vienna may be underrepresented in terms of its multicultural background compared to other cities. In this sense, initiatives to tackle childhood obesity should involve tailored strategies considering these cultural differences and social determinants of obesity. In terms of policies, there is still a gap in defining policies that target the marketing of foods to children, although, since 2017, policies to reduce physical inactivity and unhealthy diets have been launched [2].

This study showed rising levels of overweight/obesity during the first pandemic period, as reported in a southern Austrian city [34] and worldwide [13]. Indeed, previous reports from the EDDY study showed that during the onset of the pandemic, children’s body weight increased by about 4.5 kg, whereas the previous year, the average increase in body weight was below 3 kg [15]. Moreover, during the strictest period of the lockdown, boys increased 1.67 kg more than girls [35], a fact that was also observed in Klagenfurt, Austria, where boys’ increase in BMI SD scores (0.23; 95% CI, 0.18–0.29) was greater than among girls (0.09; 95% CI, 0.04–0.15) [36]. As the COVID-19 pandemic progressed, the percentage of overweight and obesity in our study sample decreased, as observed by lower rates in the year 2021. Similar results were observed in another study, where BMI SD score values stabilized during the second period of the pandemic (2020–2021) [37].

The results from our study need to be interpreted cautiously since each year’s sample size was small, particularly when analyzing by sex separately. Moreover, in 2021, the low participation rate due to the COVID-19 pandemic resulted in an even smaller sample. Using a small sample size may have increased the chance of assumptions that overweight and obesity trends were not significant, as real variability in the percentages of overweight and obesity might not be captured. It is important, therefore, that future studies consider larger samples during longer follow-up periods to render the research more efficient in terms of trends of childhood obesity in Austria. Considering the overall sample size of the children involved, our results shall not be interpreted as representative of the entire schoolchildren population of Austria. Another limitation of our study is not using linked samples from longitudinal measurements to study trends; however, we restricted the analysis to a specific school and grade each year to provide a more homogeneous group. Through this, we ensured that the contextual factors were the same. Although selecting participants during the same season was not possible, we used consistent and standardized measurement methods for assessing obesity. On the other hand, the strengths of our study are the direct measurements of weight and height.

## 5. Conclusions

In conclusion, our study showed a non-significant trend in the percentage of overweight and obesity among a sample of Viennese schoolchildren from 2017 to 2023. Nonetheless, the current prevalence of obesity remains high, with the highest peak observed in 2020, during the pandemic period. These findings emphasize the need for future investigations considering the representativeness of the school-aged population in Austria to gain a broader picture of overweight and obesity trends, as this remains a public health concern, as depicted by the current rates.

## Figures and Tables

**Table 1:** General characteristics of the participants (N = 326).

Investigation Year	N (%)	Sex (N, %)		Age	BMI (Median, 95% CI)	
		Female	Male	Median (95% CI)	Female	Male
2017	98	44 (44.9)	54 (55.1)	9.1 (8.3–10.5)	18.8 (14.6–25.9)	20.6 (14.7–28.6)
2018	64	26 (40.6)	38 (59.4)	9.0 (8.4–9.9)	18.4 (14.3–24.6)	18.1 (13.9–27.4)
2020	46	20 (43.5)	26 (56.5)	9.6 (8.8–10.5)	19.6 (16.3–28.6)	21.2 (15.1–28.2)
2021	30	17 (56.7)	13 (43.3)	9.3 (8.5–10.5)	16.5 (13.6–27.3)	19.4 (14.3–27.9)
2022	44	17 (38.6)	27 (61.4)	9.6 (8.8–10.5)	18.1 (14.3–24.2)	19.2 (14.3–29.1)
2023	44	21 (47.7)	23 (52.3)	8.9 (8.1–10.5)	18.3 (14.7–25.1)	17.2 (14.3–27.0)
Total	326	145 (44.5)	181 (55.5)	9.3 (8.3–10.5)	18.5 (14.6–25.9)	19.2 (14.3–28.1)
*p*-value *		0.70		<0.00010	0.11	0.23

* Chi-square test or Kruskal–Wallis test. BMI: body mass index, CI: confidence interval.

**Table 2:** Percentage of overweight and obesity, 2017–2023.

		Percentage (%)		
Investigation Year	N	Overweight/Obesity (95% CI)	N	Obesity (95% CI)
2017	42	42.9 (33.1–52.7)	23	23.5 (15.1–31.9)
2018	19	29.7 (18.5–40.9)	14	21.9 (11.8–32.0)
2020	23	50.0 (35.6–64.4)	13	28.3 (15.3–41.3)
2021	11	36.7 (19.5–53.9)	5	16.7 (3.4–30.0)
2022	15	34.1 (20.1–48.1)	6	13.6 (3.5–23.7)
2023	14	31.8 (18.0–45.6)	11	25.0 (12.2–37.8)
*p*-value for trend *		0.38		0.61

* Logistic regression models were applied to test the *p*-value for trends using the study year and age as continuous variables.

**Table 3:** Percentage trends in overweight and obesity from 2017 to 2023 and from 2018 to 2020.

	Overweight/Obesity	Obesity	Overweight/Obesity	Obesity
	Change from 2017–2023 ^1^ (95% CI)	Change from 2017–2023 ^1^ (95% CI)	Change from 2018–2020 ^2^ (95% CI)	Change from 2018–2020 ^2^ (95% CI)
All	−25.9% (−59.5–15.6)	6.4% (−51.2–94.9)	68.4% (5.6–187.9)	29.2% (−37.3–166.8)
Female	22.0% (−56.3–173.9)	265.9% (16.7 ± ∞) *	108% (−20.6 ± ∞) *	30.4% (−∞ ± ∞) *
Male	−45.3% (−79.6–−4.9)	−50.6% (−94.7–7.7)	57.0% (−7.7–188.0)	32.8% (−39.0–193.2)

^1^ Change from 2017 to 2023 (%): (percentage 2023 − percentage 2017)/percentage 2017 × 100. ^2^ Change from 2018 to 2020 (%): (percentage 2020 − percentage 2018)/percentage 2018 × 100. * These confidence intervals suggest that there is no upper (or lower) limit to the proportion of participants with overweight or obesity.

## Data Availability

The data presented in this study are available on request from the corresponding author due to ethical reasons.

## Supplementary Materials

The following supporting information can be downloaded at: https://www.mdpi.com/article/10.3390/children11040431/s1, Table S1: The differences between the participants included in the study and those excluded; Table S2: Percentage (95% CI) of overweight/obesity and obesity according to age and sex; Table S3: Percentage of overweight and obesity by sex, 2017–2023.
