# Supplementary material for: Childhood Obesity Trends among 8–11-Year-Olds: Insights from a School Sample in Vienna, Austria (2017–2023)
Source: Children (Basel). 2024 Apr 3;11(4):431. doi: 10.3390/children11040431 (PMC11049460; doi:10.3390/children11040431)
Supplement: Supplementary file 1 [file children-11-00431-s001.zip › children-2920989-supplementary.pdf]

children

MDPI

Article

Childhood Obesity Trends among 8–11-Year-Olds: Insights from a School Sample in Vienna, Austria (2017–2023)

Supplementary Materials

Table S1. The differences between the participants included in the study and those excluded.

Variable	2017	2018	2020	2021	2022	*p*-value*
**N (%)**						
included	98	64	46	30	44	0.014
excluded	45	60	38	35	34	
**Female**						
included	44 (67.7)	26 (50.0)	20 (48.8)	17 (43.6)	17 (45.9)	0.083
excluded	21 (32.3)	26 (50.0)	21 (51.2)	22 (56.4)	20 (54.1)	
**Age (years)**						
included	9.1 (8.25-10.5)	9.0 (8.4-9.9)	9.6 (8.8-10.5)	9.3 (8.5-10.5)	9.6 (8.8-10.5)	<0.0001
excluded	9.8 (9.1-10.3)	10 (9.4-10.7)	10.4 (9.9-10.8)	10.1 (9.4-10.8)	10.4 (9.8-10.8)	
**BMI (kg/m²)**						
included	19.3 (14.7-28.5)	18.2 (14.1-25.5)	20.3 (15.2-28.2)	17.2 (14.3-27.3)	18.6 (14.3-27.1)	0.058
excluded	18.0 (14.7-26.1)	20.0 (15.1-30.6)	22.4 (14.9-29.6)	20.0 (15.1-27.9)	19.7 (14.1-31.0)	

* The Chi-square test or Kruskal–Wallis test was used. BMI: body mass index. Values show the median and 95% confidence interval (CI). Exclusion because they were part of the intervention group. The year 2023 was not included, as a change in the study methodology determined that children from 4th grade were not measured.

Table S2. Percentage (95% CI) of overweight/obesity and obesity according to age and sex.

	Percentage overweight/obesity			Percentage obesity		
**Female/Age**	N	% (95% CI)	*p*-value*	N	% (95% CI)	*p*-value*
8	16	27.6 (5.7-49.5)	0.057	8	13.8 (0-37.7)	0.027
9	17	23.6 (3.4-43.8)	0.0031	8	11.1 (0-32.9)	0.0054
10	6	40.0 (0.8-79.2)	0.34	4	26.7 (0-71.4)	0.85
**Male/Age**						
8	23	45.1 (24.8-65.4)		16	31.4 (8.7-54.1)	
9	46	45.5 (31.1-59.9)		29	28.7 (12.2-45.2)	
10	16	55.2 (30.8-79.6)		7	24.1 (0-55.8)	

* *p*-value for the difference between sexes using the Chi-square test.

Table S3. Percentage of overweight and obesity by sex, 2017–2023.

Investigation year	N	Female % (95% CI)	N	Male % (95% CI)
**Percentage overweight/obesity**				
2017	12	27.3 (14.1-40.5)	30	55.6 (42.3-68.9)
2018	5	19.2 (4.1-34.3)	14	36.8 (21.5-52.1)
2020	8	40.0 (18.5-61.5)	15	57.7 (38.7-76.7)
2021	4	23.5 (3.3-43.7)	7	53.9 (26.8-81.0)
2022	3	17.7 (0-35.8)	12	44.4 (25.7-63.1)
2023	7	33.3 (12.9-53.1)	7	30.4 (11.6-49.2)
p-value for trend*		0.75		0.20
**Percentage obesity**				
2017	4	9.1 (0.6-17.6)	19	35.2 (22.5-47.9)
2018	3	11.5 (0-23.8)	11	29.0 (14.6-43.4)
2020	3	15.0 (0-30.6)	10	38.5 (19.8-57.2)
2021	2	11.8 (0-27.1)	3	23.1 (0.2-46.0)
2022	1	5.9 (0-17.1)	5	18.5 (3.9-33.1)
2023	7	33.3 (13.1-53.5)	4	17.4 (1.9-32.9)
p-value for trend*		0.082		0.094

*Logistic regression models were applied to test the *p*-value for trends using the study year and age as continuous variables.
